# Association between genetic polymorphisms and endometrial cancer risk: a systematic review

**DOI:** 10.1136/jmedgenet-2019-106529

**Published:** 2020-02-17

**Authors:** Cemsel Bafligil, Deborah J Thompson, Artitaya Lophatananon, Miriam J Smith, Neil AJ Ryan, Anie Naqvi, D Gareth Evans, Emma J Crosbie

**Affiliations:** 1 Division of Cancer Sciences, University of Manchester, Manchester, UK; 2 Department of Public Health and Primary Care, University of Cambridge, Cambridge, UK; 3 Division of Population Health, University of Manchester, Manchester, UK; 4 Division of Evolution and Genomic Sciences, University of Manchester, Manchester, UK; 5 University of Manchester Medical School, Manchester, UK; 6 Department of Obstetrics and Gynaecology, Manchester University NHS Foundation Trust, Manchester, UK

**Keywords:** endometrial cancer, single nucleotide polymorphism (SNP), systematic review, genetic epidemiology, risk prediction

## Abstract

**Introduction:**

Endometrial cancer is one of the most commonly diagnosed cancers in women. Although there is a hereditary component to endometrial cancer, most cases are thought to be sporadic and lifestyle related. The aim of this study was to systematically review prospective and retrospective case–control studies, meta-analyses and genome-wide association studies to identify genomic variants that may be associated with endometrial cancer risk.

**Methods:**

We searched MEDLINE, Embase and CINAHL from 2007 to 2019 without restrictions. We followed PRISMA 2009 guidelines. The search yielded 3015 hits in total. Following duplicate exclusion, 2674 abstracts were screened and 453 full-texts evaluated based on our pre-defined screening criteria. 149 articles were eligible for inclusion.

**Results:**

We found that single nucleotide polymorphisms (SNPs) in *HNF1B*, *KLF*, *EIF2AK*, *CYP19A1*, *SOX4* and *MYC* were strongly associated with incident endometrial cancer. Nineteen variants were reported with genome-wide significance and a further five with suggestive significance. No convincing evidence was found for the widely studied *MDM2* variant rs2279744. Publication bias and false discovery rates were noted throughout the literature.

**Conclusion:**

Endometrial cancer risk may be influenced by SNPs in genes involved in cell survival, oestrogen metabolism and transcriptional control. Larger cohorts are needed to identify more variants with genome-wide significance.

## Introduction

Endometrial cancer is the most common gynaecological malignancy in the developed world.^1^ Its incidence has risen over the last two decades as a consequence of the ageing population, fewer hysterectomies for benign disease and the obesity epidemic. In the USA, it is estimated that women have a 1 in 35 lifetime risk of endometrial cancer, and in contrast to cancers of most other sites, cancer-specific mortality has risen by approximately 2% every year since 2008 related to the rapidly rising incidence.^2^

Endometrial cancer has traditionally been classified into type I and type II based on morphology.^3^ The more common subtype, type I, is mostly comprised of endometrioid tumours and is oestrogen-driven, arises from a hyperplastic endometrium, presents at an early stage and has an excellent 5 year survival rate.^4^ By contrast, type II includes non-endometrioid tumours, specifically serous, carcinosarcoma and clear cell subtypes, which are biologically aggressive tumours with a poor prognosis that are often diagnosed at an advanced stage.^5^ Recent efforts have focused on a molecular classification system for more accurate categorisation of endometrial tumours into four groups with distinct prognostic profiles.^6 7^

The majority of endometrial cancers arise through the interplay of familial, genetic and lifestyle factors. Two inherited cancer predisposition syndromes, Lynch syndrome and the much rarer Cowden syndrome, substantially increase the lifetime risk of endometrial cancer, but these only account for around 3–5% of cases.^8–10^ Having first or second degree relative(s) with endometrial or colorectal cancer increases endometrial cancer risk, although a large European twin study failed to demonstrate a strong heritable link.^11^ The authors failed to show that there was greater concordance in monozygotic than dizygotic twins, but the study was based on relatively small numbers of endometrial cancers. Lu and colleagues reported an association between common single nucleotide polymorphisms (SNPs) and endometrial cancer risk, revealing the potential role of SNPs in explaining part of the risk in both the familial and general populations.^12^ Thus far, many SNPs have been reported to modify susceptibility to endometrial cancer; however, much of this work predated genome wide association studies and is of variable quality. Understanding genetic predisposition to endometrial cancer could facilitate personalised risk assessment with a view to targeted prevention and screening interventions.^13^ This emerged as the most important unanswered research question in endometrial cancer according to patients, carers and healthcare professionals in our recently completed James Lind Womb Cancer Alliance Priority Setting Partnership.^14^ It would be particularly useful for non-endometrioid endometrial cancers, for which advancing age is so far the only predictor.^15^

We therefore conducted a comprehensive systematic review of the literature to provide an overview of the relationship between SNPs and endometrial cancer risk. We compiled a list of the most robust endometrial cancer-associated SNPs. We assessed the applicability of this panel of SNPs with a theoretical polygenic risk score (PRS) calculation. We also critically appraised the meta-analyses investigating the most frequently reported SNPs in *MDM2*. Finally, we described all SNPs reported within genes and pathways that are likely involved in endometrial carcinogenesis and metastasis.

## Methods

Our systematic review follows the Preferred Reporting Items for Systematic Reviews and Meta-Analyses (PRISMA) collaboration 2009 recommendations. The registered protocol is available through PROSPERO (CRD42018091907).^16^

### Search strategy

We searched Embase, MEDLINE and Cumulative Index to Nursing and Allied Health Literature (CINAHL) databases via the Healthcare Databases Advanced Search (HDAS) platform, from 2007 to 2018, to identify studies reporting associations between polymorphisms and endometrial cancer risk. Key words including MeSH (Medical Subject Heading) terms and free-text words were searched in both titles and abstracts. The following terms were used: “endomet*”,“uter*”, “womb”, “cancer(s)”, “neoplasm(s)”, “endometrium tumour”, “carcinoma”, “adenosarcoma”, “clear cell carcinoma”, “carcinosarcoma”, “SNP”, “single nucleotide polymorphism”, “GWAS”, and “genome-wide association study/ies”. No other restrictions were applied. The search was repeated with time restrictions between 2018 and June 2019 to capture any recent publications.

### Eligibility criteria

Studies were selected for full-text evaluation if they were primary articles investigating a relationship between endometrial cancer and SNPs. Study outcome was either the increased or decreased risk of endometrial cancer relative to controls reported as an odds ratio (OR) with corresponding 95% confidence intervals (95% CIs).

### Study selection

Three independent reviewers screened all articles uploaded to a screening spreadsheet developed by Helena VonVille.^17^ Disagreements were resolved by discussion. Chronbach’s α score was calculated between reviewers and indicated high consistency at 0.92. Case–control, prospective and retrospective studies, genome-wide association studies (GWAS), and both discovery and validation studies were selected for full-text evaluation. Non-English articles, editorials, conference abstracts and proceedings, letters and correspondence, case reports and review articles were excluded.

Candidate-gene studies with at least 100 women and GWAS with at least 1000 women in the case arm were selected to ensure reliability of the results, as explained by Spencer *et al*.^18^ To construct a panel of up to 30 SNPs with the strongest evidence of association, those with the strongest p values were selected. For the purpose of an SNP panel, articles utilising broad European or multi-ethnic cohorts were selected. Where overlapping populations were identified, the most comprehensive study was included.

### Data extraction and synthesis

For each study, the following data were extracted: SNP ID, nearby gene(s)/chromosome location, OR (95% CI), p value, minor or effect allele frequency (MAF/EAF), EA (effect allele) and OA (other allele), adjustment, ethnicity and ancestry, number of cases and controls, endometrial cancer type, and study type including discovery or validation study and meta-analysis. For risk estimates, a preference towards most adjusted results was applied. For candidate-gene studies, a standard p value of<0.05 was applied and for GWAS a p value of <5×10^-8^, indicating genome-wide significance, was accepted as statistically significant. However, due to the limited number of SNPs with p values reaching genome-wide significance, this threshold was then lowered to <1×10^-5^, allowing for marginally significant SNPs to be included. As shown by Mavaddat *et al,* for breast cancer, SNPs that fall below genome-wide significance may still be useful for generating a PRS and improving the models.^19^

We estimated the potential value of a PRS based on the most significant SNPs by comparing the predicted risk for a woman with a risk score in the top 1% of the distribution to the mean predicted risk. Per-allele ORs and MAFs were taken from the publications and standard errors (SEs) for the lnORs were derived from published 95% CIs. The PRS was assumed to have a Normal distribution, with mean 2∑β_i_p_I_ and SE, σ, equal to √2∑β_i_^2^p_I_(1−p_i_), according to the binomial distribution, where the summation is over all SNPs in the risk score. Hence the relative risk (RR) comparing the top 1% of the distribution to the mean is given by exp(Z_0.01_σ), where Z is the inverse of the standard normal cumulative distribution.

## Results

**Figure 1:**
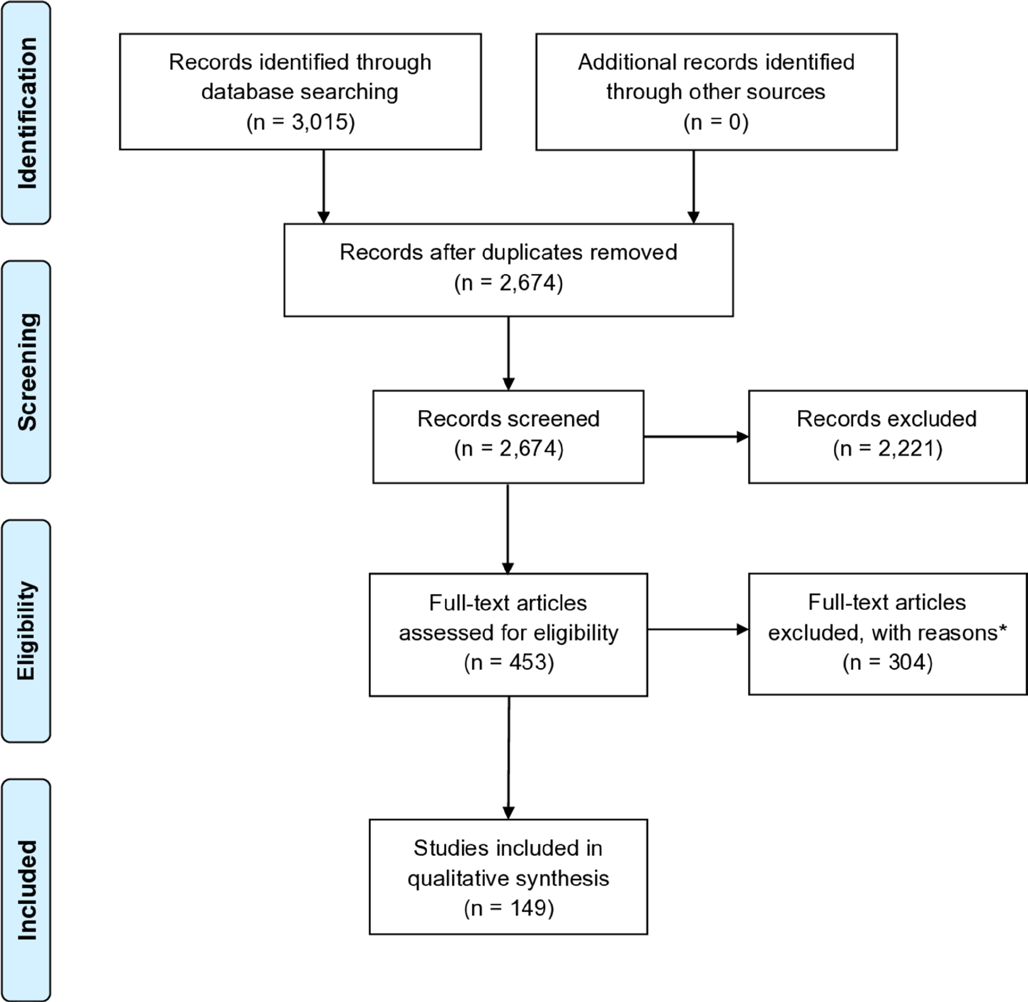
Study selection flow diagram. *Reasons: irrelevant articles, articles focusing on other conditions, non-GWAS/candidate-gene study related articles, technical and duplicate articles. GWAS, genome-wide association study. Adapted from: Moher D, Liberati A, Tetzlaff J, Altman DG, The PRISMA Group (2009). Preferred Reporting Items for Systematic Reviews and Meta-Analyses: The PRISMA Statement. *PLoS Med* 6(6): e1000097. doi:10.1371/journal.pmed1000097.

The flow chart of study selection is illustrated in figure 1. In total, 453 text articles were evaluated and, of those, 149 articles met our inclusion criteria. One study was excluded from table 1, for having an Asian-only population, as this would make it harder to compare with the rest of the results which were all either multi-ethnic or Caucasian cohorts, as stated in our inclusion criteria for the SNP panel.^20^ Any SNPs without 95% CIs were also excluded from any downstream analysis. Additionally, SNPs in linkage disequilibrium (r^2^ >0.2) with each other were examined, and of those in linkage disequilibrium, the SNP with strongest association was reported. Per allele ORs were used unless stated otherwise.

**Table 1:** List of top SNPs most likely to contribute to endometrial cancer risk identified through systematic review of recent literature^21–25^

Reference	SNP ID	Nearby gene(s)	Location	OR	LCI	UCI	P	EAF	EA	OA	Ethnicity	Cases (n)	Controls (n)	EC type	Position	Datasets*
O'Mara *et al*, 2018^21^	rs11263761	HNF1B	17q12	1.15	1.12	1.19	3.20e-20	0.52	A	G	EUR	12 906	108 979	All	Intronic	**NSECG**, UK1-CORGI, **SEARCH**, WTCCC, **ANECS**, QIMR, HCS, E2C2, BECS/HJECS, **iCOGS**† (BBCC, BSUCH, ESTHER, GC-HBOC, GENICA, MARIE, **MoMaTEC**, NBCS, BBCS, SBCS, UKBGS), **NECS**, ABCFS, ABCTB, BCEES, MCCS, **LES**, LMBC, **BECS**, GESBC, HaBCS, CAHRES, RENDOCAS, MISS, pKARMA, SMC, CBR_STUDY98, MECS, MCBCS, MMHS, WHI, UKBB
	rs7981863	KLF5, KLF12	13q22.1	1.16	1.12	1.20	2.70e-17	0.72	C	T	EUR	12 906	108 979	All	Intergenic	
	rs1740828	SOX4	6p22.3	1.15	1.11	1.19	4.20e-16	0.52	G	A	EUR	12 906	108 979	All	Regulatory region	
	rs17601876	CYP19A1	15q21.2	1.12	1.09	1.16	3.30e-14	0.48	G	A	EUR	12 906	108 979	All	Intronic	
	rs4733613	MYC	8q24.21	1.18	1.13	1.24	7.50e-14	0.12	C	G	EUR	12 906	108 979	All	Intergenic	
	rs3184504	SH2B3	12q24.11	1.10	1.07	1.14	1.10e-10	0.52	C	T	EUR	12 906	108 979	All	Missense	
	rs2747716	HEY2, NCOA7, MYC	6q22.31	1.10	1.07	1.14	2.90e-10	0.57	A	G	EUR	12 906	108 979	All	Intronic	
	rs9668337	SSPN	12p12.1	1.11	1.08	1.15	1.10e-09	0.74	A	G	EUR	12 906	108 979	All	Non-coding exon	
	rs35286446	MYC	8q24.21	1.10	1.06	1.13	3.10e-09	0.58	GAT	G	EUR	12 906	108 979	All	Intronic	
	rs10850382	LOC107984437	12q24.21	1.10	1.07	1.14	3.50e-09	0.31	T	C	EUR	12 906	108 979	All	Regulatory region	
	rs882380	SKAP, SNX11	17q21.32	1.10	1.06	1.13	4.70e-09	0.61	A	C	EUR	12 906	108 979	All	Intronic	
	rs937213	EIF2AK4, BMF	15q15.1	1.09	1.06	1.13	5.10e-09	0.42	C	T	EUR	12 906	108 979	All	Intronic	
	rs1679014	CDKN2A, CDKN2B	9p21.3	1.18	1.12	1.25	6.40e-09	0.07	T	C	EUR	12 906	108 979	All	Intronic	
Painter *et al*, 2016^22^	rs2498794	AKT1	14q32.33	1.13	1.09	1.17	8.70e-09	0.48	G	A	EUR	7737	37 144	All	Intronic	**ANECS**, **SEARCH**, **NSECG**, **iCOGS**
O'Mara *et al*, 2018^21^	rs10835920	WT1, WT1-AS, EIF3M	11p13	1.09	1.06	1.13	1.30e-08	0.38	T	C	EUR	12 906	108 979	All	Intergenic	**NSECG**, UK1-CORGI, **SEARCH**, WTCCC, **ANECS**, QIMR, HCS, E2C2, BECS/HJECS, **iCOGS**, BBCC, BSUCH, ESTHER, GC-HBOC, GENICA, MARIE, **MoMaTEC**, NBCS, BBCS, SBCS, UKBGS, **NECS**, ABCFS, ABCTB, BCEES, MCCS, **LES**, LMBC, **BECS**, GESBC, HaBCS, CAHRES, RENDOCAS, MISS, pKARMA, SMC, CBR_STUDY98, MECS, MCBCS, MMHS, WHI, UKBB
	rs139584729	MYC	8q24.21	1.40	1.25	1.58	2.40e-08	0.98	C	G	EUR	12 906	108 979	All	Intergenic	
	rs148261157	BCL11A	2p16.1	1.26	1.16	1.36	3.40e-08	0.03	A	G	EUR	12 906	108 979	All	Intergenic	
				1.25	1.14	1.38	4.70e-06	0.03				8758	46 126	Endometrioid		
				1.64	1.32	2.04	9.60e-06	0.03				1230	35 447	Non-endometrioid		
	rs113998067	GNL2, RSPO1, CDCA8	1p34.3	1.23	1.14	1.32	3.60e-08	0.04	C	T	EUR	12 906	108 979	All	Intergenic	
	rs1129506	EVI2A, NF1	17q11.2	1.10	1.06	1.13	4.30e-08	0.38	G	A	EUR	12 906	108 979	All	Missense	
Spurdle *et al*, 2011^23^	rs673604	SFPQ	1p34	1.21	1.12	1.32	5.90e-06	0.08	G	A	EUR	1265	5190	All	Regulatory region	**ANECS**, **NECS**, **SEARCH**, WTCCC2, **BECS**, **LES**, **MoMaTEC**, **NSECG**, PECS, SASBAC, SECGS
O'Mara *et al*, 2015^24^	rs79575945	ESR1	6q25	1.20	1.11	1.30	3.76e-06	0.07	G	A	EUR	6607	37 925	Endometrioid	Intronic	**iCOGS**, BCAC, OCAC, **ANECS**, **SEARCH**, **NSECG**
Chen *et al*, 2014^25^	rs1953358	LINC00520	14q22.3	1.36	1.20	1.53	4.76e-07	0.49	G	A	ME	1055	1778	Endometrioid	Intergenic	AHS, EDGE, FHCRC, MEC
	rs8178648	PROS1	chr3	1.71	1.37	2.12	1.53e-06	0.09	G	A	ME	1055	1778	Endometrioid	Intronic	
	rs9399840	N/A	6q16.3	1.33	1.18	1.49	3.01e-06	0.53	T	C	ME	1055	1778	Endometrioid	Intergenic	

The different studies listed here used overlapping datasets (in bold).

All locations were based on Genome Reference Consortium Human Build 37 (GRCh37). Variants located at 8q24.21 were obtained from a conditional model

*NSECG: UK National Study of Endometrial Cancer Genetics, UK1-CORGI: UK Colorectal Tumour Gene Identification Consortium, SEARCH: UK Studies of Epidemiology and Risk factors in Cancer Heredity, WTCCC1/2: Wellcome Trust Case Control Consortium 1/2, ANECS: Australian National Endometrial Cancer Study, QIMR: Queensland Institute of Medical Research, HCS: Hunter Community Study, E2C2: NCI-supported international consortium of four US-based cohort studies, 2 US-based case-control studies and 1 Polish case-control study, BECS/HJECS: Bavarian Endometrial Cancer Study/Hannover-Jena Endometrial Cancer Study, BBCC: Bavarian Breast Cancer Cases and Controls, BSUCH: Breast Cancer Study of the University Clinic Heidelberg, ESTHER: ESTHER Breast Cancer Study, GC-HBOC: German Consortium for Hereditary Breast & Ovarian Cancer, GENICA: Gene Environment Interaction and Breast Cancer in Germany, MARIE: Mammary Carcinoma Risk Factor Investigation, MoMaTEC: Molecular Markers in Treatment of Endometrial Cancer, NBCS: Norwegian Breast Cancer Study, SEARCH: UK Studies of Epidemiology and Risk factors in Cancer Heredity, NSECG: National Study of Endometrial Cancer Genetics, BBCS: British Breast Cancer Study, SBCS: Sheffield Breast Cancer Study, UKBGS: UK Breakthrough Generations Study, ANECS: Australian National Endometrial Cancer Study, NECS: Newcastle Endometrial Cancer Study, ABCFS: Australian Breast Cancer Family Study, ABCTB: Australian Breast Cancer Tissue Bank, BCEES: Breast Cancer Employment and Environment Study, MCCS: Melbourne Collaborative Cohort Study, LES: Leuven Endometrial Cancer Study, LMBC: Leuven Multidisciplinary Breast Centre, BECS: Bavarian Endometrial Cancer Study, BBCC: Bavarian Breast Cancer Cases and Controls, BSUCH: Breast Cancer Study of the University Clinic Heidelberg, GENICA: Gene Environment Interaction and Breast Cancer in Germany, GESBC: Genetic Epidemiology Study of Breast Cancer by Age 50, HaBCS: Hannover Breast Cancer Study, MARIE: Mammary Carcinoma Risk Factor Investigation, CAHRES: Cancer Hormone Replacement Epidemiology, RENDOCAS: Registry of Endometrial Cancer in Sweden, MISS: Melanoma Inquiry of Southern Sweden, pKARMA: Karolinska Mammography Project for Risk Prediction of Breast Cancer, SMC: Swedish Mammography Cohort, SEARCH: UK Studies of Epidemiology and Risk factors in Cancer Heredity, NSECG: National Study of Endometrial Cancer Genetics, BBCS: British Breast Cancer Study, CBR_STUDY98: Cambridge BioResource, UKBGS: UK Breakthrough Generations Study, MECS: Mayo Endometrial Cancer Study, MCBCS: Mayo Clinic Breast Cancer Study, MMHS: Mayo Mammography Health Study, WHI: Women's Health Initiative, UKBB: UK BioBank, OCAC: Ovarian Cancer Association Consortium, AHS: Alberta Health Services, EDGE: Oestrogen, Diet, Genetics and Endometrial Cancer, FHCRC: Fred Hutchinson Cancer Research Centre, MEC: Multiethnic Cohort Study, PECS: Polish Endometrial Cancer Study, SASBAC: Singapore and Swedish Breast/Endometrial Cancer Study, SECGS: Shanghai Endometrial Cancer Genetic Study.

†iCOGS dataset breakdown was not indicated by the listed studies here other than O’Mara *et al*., 2018.^21^

EA, effect allele; EAF, effect allele frequency; EC, endometrial cancer; EUR, European cohort; LCI, lower confidence interval; ME, multiethnic; OA, other allele; SNP, single nucleotide polymorphism; UCI, upper confidence interval.

### Top SNPs associated with endometrial cancer risk

Following careful interpretation of the data, 24 independent SNPs with the lowest p values that showed the strongest association with endometrial cancer were obtained (table 1).^21–25^ These SNPs are located in or around genes coding for transcription factors, cell growth and apoptosis regulators, and enzymes involved in the steroidogenesis pathway. All the SNPs presented here were reported on the basis of a GWAS or in one case, an exome-wide association study, and hence no SNPs from candidate-gene studies made it to the list. This is partly due to the nature of larger GWAS providing more comprehensive and powered results as opposed to candidate gene studies. Additionally, a vast majority of SNPs reported by candidate-gene studies were later refuted by large-scale GWAS such as in the case of *TERT* and *MDM2* variants.^26 27^ The exception to this is the *CYP19* gene, where candidate-gene studies reported an association between variants in this gene with endometrial cancer in both Asian and broad European populations, and this association was more recently confirmed by large-scale GWAS.^21 28–30^ Moreover, a recent article authored by O’Mara and colleagues reviewed the GWAS that identified most of the currently known SNPs associated with endometrial cancer.^31^

Most of the studies represented in table 1 are GWAS and the majority of these involved broad European populations. Those having a multi-ethnic cohort also consisted primarily of broad European populations. Only four of the variants in table 1 are located in coding regions of a gene, or in regulatory flanking regions around the gene. Thus, most of these variants would not be expected to cause any functional effects on the gene or the resulting protein. An eQTL search using GTEx Portal showed that some of the SNPs are significantly associated (p<0.05) with modified transcription levels of the respective genes in various tissues such as prostate (rs11263761), thyroid (rs9668337), pituitary (rs2747716), breast mammary (rs882380) and testicular (rs2498794) tissue, as summarised in table 2.

**Table 2:** List of eQTL hits for the selected panel of SNPs

SNP ID	Significant eQTL for	P	Tissue	Other gene(s)	Other tissue(s)
rs17601876	GLDN	1.2e-08	Adipose – subcutaneous	SPPL2A, DMXL2	Skin – sun exposed (lower leg); colon – sigmoid; cells – cultured fibroblasts; muscle – skeletal; spleen; skin – not sun exposed (suprapubic); nerve – tibial
	CYP19A1	3.4e-07	Whole blood		
	CYP19A1	5.8e-06	Adipose – subcutaneous		
rs3184504	TMEM116	1.7e-04	Adipose – subcutaneous	ALDH2, LINC01405, ADAM1B	Oesophagus – mucosa; skin – not sun exposed (suprapubic); skin – sun exposed (lower leg); muscle – skeletal; artery – aorta; heart – atrial appendage; artery – tibial; colon – sigmoid; brain – nucleus accumbens (basal ganglia)
	MAPKAPK5	2.6e-04	Adipose – subcutaneous		
rs2747716	RP11-624M8.1	4.2e-11	Pituitary	HDDC2	Artery – tibial; pancreas; thyroid; brain – nucleus accumbens (basal ganglia); brain – substantia nigra; oesophagus – muscularis; nerve – tibial; Brain – caudate (basal ganglia); adipose – visceral (omentum); brain – spinal cord (cervical c-1); artery – aorta; brain – cortex; brain – hypothalamus; muscle – skeletal; brain – cerebellum; heart – left ventricle; brain – putamen (basal ganglia); brain – frontal cortex (BA9); brain – cerebellar hemisphere
	RP11-624M8.1	8.2e-11	Adipose – subcutaneous		
	HEY2	9.7e-10	Testis		
	HEY2	2.1e-09	Ovary		
	RP11-624M8.1	1.7e-07	Breast – mammary tissue		
	RP11-624M8.1	1.3e-06	Ovary		
rs9668337	BHLHE41	9.0e-17	Thyroid	RP11-283G6.3	Cells – cultured fibroblasts
	SSPN	1.1e-04	Thyroid		
rs882380	SNX11	3.1e-25	Adipose – subcutaneous	RP5-890E16.5, CBX1, LRRC46, MRPL10, RP11-6N17.4, CDK5RAP3, SP6, PRR15L, RP5-890E16.2, PNPO, RP11-6N17.3, HOXB1, HOXB-AS1, NFE2L1	Skin – sun exposed (lower leg); cells – cultured fibroblasts; adipose – visceral (omentum); lung; skin – not sun exposed (suprapubic); pancreas; spleen; oesophagus – muscularis; artery – aorta; heart – atrial appendage; liver; colon – transverse; thyroid; artery – tibial; colon – sigmoid; oesophagus – gastro-oesophageal junction; stomach; muscle – skeletal; small intestine – terminal Ileum; prostate; brain – cerebellum; brain – cerebellar hemisphere; minor salivary gland; adrenal gland; oesophagus – mucosa
	SNX11	1.0e-21	Whole blood		
	SNX11	1.2e-13	Breast – mammary tissue		
	COPZ2	9.3e-12	Testis		
	SKAP1	3.3e-08	Whole blood		
	HOXB2	2.6e-05	Adipose – subcutaneous		
rs937213	EIF2AK4	4.7e-11	Adipose – visceral (omentum)	SRP14	Thyroid; oesophagus – mucosa; skin – sun exposed (lower leg); stomach; oesophagus – muscularis; pancreas; skin – not sun exposed (suprapubic); colon – transverse; adipose – subcutaneous; lung; colon – sigmoid; muscle – skeletal; nerve – tibial; whole blood; oesophagus – gastro-oesophageal junction; artery – tibial; adrenal gland; spleen; heart – left ventricle; heart – atrial appendage
	EIF2AK4	3.4e-08	Breast – mammary tissue	N/A	
	RP11-521C20.5	5.4e-07	Testis	N/A	
	RP11-521C20.5	7.4e-07	Prostate	N/A	
rs2498794	AKT1	1.7e-30	Thyroid	ZBTB42	Oesophagus – mucosa; artery – tibial; oesophagus – muscularis; skin – sun exposed (lower leg); skin – not sun exposed (suprapubic); cells – cultured fibroblasts; artery – aorta; oesophagus – gastro-oesophageal junction; adipose – subcutaneous; colon – sigmoid; colon – transverse; heart – atrial appendage
	ADSSL1	5.5e-25	Testis		
	SIVA1	1.8e-07	Adipose – visceral (omentum)		
	ADSSL1	2.6e-05	Ovary		
	SIVA1	4.4e-05	Breast – mammary tissue		
rs10835920	WT1-AS	5.5e-06	Spleen	N/A	Oesophagus -– muscularis
rs148261157	KIAA1841	1.3e-05	Oesophagus – muscularis	N/A	N/A
rs113998067	RSPO1	2.7e-10	Artery – tibial	EPHA10, FHL3, DNALI1	Nerve – tibial; artery – aorta; colon – transverse
rs1129506	EVI2A	4.3e-20	Whole blood	OMG, RAB11FIP4	Spleen; oesophagus – mucosa; artery – tibial; lung; artery – aorta; skin – sun exposed (lower leg); nerve – tibial; heart – atrial appendage; adipose – visceral (omentum); cells – cultured fibroblasts; liver; stomach; brain – amygdala; skin – not sun exposed (suprapubic); brain – caudate (basal ganglia); muscle – skeletal; colon – sigmoid
	NF1	3.5e-09	Adipose – subcutaneous		
	NF1	2.2e-07	Thyroid		
	NF1	3.7e-07	Testis		
rs673604	ZMYM1	7.0e-07	Adipose – subcutaneous	RP4-665N4.8, ZMYM4, KIAA0319L, TFAP2E	Skin – sun exposed (lower leg); oesophagus – muscularis; cells – EBV-transformed lymphocytes; oesophagus – mucosa; nerve – tibial; brain – cerebellum
	MAP7D1	1.0e-05	Whole blood		
rs1953358	LINC00520	1.5e-05	Skin – not sun exposed (suprapubic)	N/A	N/A
rs8178648	PROS1	3.0e-04	Skin – sun exposed (lower leg)	N/A	N/A

Top significant eQTL hits from different tissues are shown in the table. There were no significant hits reported for some SNPs which are hence not included in this table.

EBV, Epstein-Barr virus; SNP, single nucleotide polymorphism.

The only variant for which there was an indication of a specific association with non-endometrioid endometrial cancer was rs148261157 near the *BCL11A* gene. The A allele of this SNP had a moderately higher association in the non-endometrioid arm (OR 1.64, 95% CI 1.32 to 2.04; p=9.6×10^-6^) compared with the endometrioid arm (OR 1.25, 95% CI 1.14 to 1.38; p=4.7×10^-6^).^21^

Oestrogen receptors α and β encoded by *ESR1* and *ESR2*, respectively, have been extensively studied due to the assumed role of oestrogens in the development of endometrial cancer. O’Mara *et al* reported a lead SNP (rs79575945) in the *ESR1* region that was associated with endometrial cancer (p=1.86×10^-5^).^24^ However, this SNP did not reach genome-wide significance in a more recent larger GWAS.^21^ No statistically significant associations have been reported between endometrial cancer and SNPs in the *ESR2* gene region.

*AKT* is an oncogene linked to endometrial carcinogenesis. It is involved in the PI3K/AKT/mTOR pro-proliferative signalling pathway to inactivate apoptosis and allow cell survival. The A allele of rs2494737 and G allele of rs2498796 were reported to be associated with increased and decreased risk of endometrial cancer in 2016, respectively.^22 30^ However, this association was not replicated in a larger GWAS in 2018.^21^ Nevertheless, given the previous strong indications, and biological basis that could explain endometrial carcinogenesis, we decided to include an *AKT1* variant (rs2498794) in our results.

*PTEN* is a multi-functional tumour suppressor gene that regulates the AKT/PKB signalling pathway and is commonly mutated in many cancers including endometrial cancer.^32^ Loss-of-function germline mutations in *PTEN* are responsible for Cowden syndrome, which exerts a lifetime risk of endometrial cancer of up to 28%.^9^ Lacey and colleagues studied SNPs in the *PTEN* gene region; however, none showed significant differences in frequency between 447 endometrial cancer cases and 439 controls of European ancestry.^33^

*KRAS* mutations are known to be present in endometrial cancer. These can be activated by high levels of KLF5 (transcriptional activator). Three SNPs have been identified in or around *KLF5* that are associated with endometrial cancer. The G allele of rs11841589 (OR 1.15, 95% CI 1.11 to 1.21; p=4.83×10^-11^), the A allele of rs9600103 (OR 1.23, 95% CI 1.16 to 1.30; p=3.76×10^-12^) and C allele of rs7981863 (OR 1.16, 95% CI 1.12 to 1.20; p=2.70×10^-17^) have all been found to be associated with an increased likelihood of endometrial cancer in large European cohorts.^21 30 34^ It is worth noting that these SNPs are not independent, and hence they quite possibly tag the same causal variant.

The *MYC* family of proto-oncogenes encode transcription factors that regulate cell proliferation, which can contribute to cancer development if dysregulated. The recent GWAS by O’Mara *et al* reported three SNPs within the *MYC* region that reached genome-wide significance with conditional p values reaching at least 5×10^–8^.^35^

To test the utility of these SNPs as predictive markers, we devised a theoretical PRS calculation using the log ORs and EAFs per SNP from the published data. The results were very encouraging with an RR of 3.16 for the top 1% versus the mean, using all the top SNPs presented in table 1 and 2.09 when using only the SNPs that reached genome-wide significance (including *AKT1*).

### Controversy surrounding *MDM2* variant SNP309

*MDM2* negatively regulates tumour suppressor gene *TP53*, and as such, has been extensively studied in relation to its potential role in predisposition to endometrial cancer. Our search identified six original studies of the association between *MDM2* SNP rs2279744 (also referred to as SNP309) and endometrial cancer, all of which found a statistically significant increased risk per copy of the G allele. Two more original studies were identified through our full-text evaluation; however, these were not included here as they did not meet our inclusion criteria—one due to small sample size, the other due to studying rs2279744 status dependent on another SNP.^36 37^ Even so, the two studies were described in multiple meta-analyses that are listed in table 3. Different permutations of these eight original studies appear in at least eight published meta-analyses. However, even the largest meta-analysis contained <2000 cases (table 3)^38^

**Table 3:** Characteristics of studies that examined *MDM2* SNP rs2279744

Reference	OR (95% CI)	P values	EAF	Ancestry	Cases (n)	Controls (n)	EC type	Dataset(s)
Terry 2008^48^	1.32 (1.11 to 1.56)	0.002	N/A	European	591	1543	N/A	NHS (Nurses’ Health Study), WHS (Women’s Health Study)
Ashton 2009^49^	1.37 (1.06 to 1.79)	N/A	0.56	Caucasian	191	291	All	Hospital based
Nunobiki 2009^50^	2.28 (2.02 to 2.54)	0.030	0.49	Japanese	102	95	All	Hospital based
Ueda 2009^51^	1.91 (1.5 to 3.47)	0.035	0.51	Japanese	119	108	All	Hospital based
Wan 2011^43^	1.54 (1.21 to 1.94)	0.000	N/A	N/A	N/A	N/A	N/A	Walsh 2007,^36^ Terry 2008, Ashton 2009, Nunobiki 2009, Ueda 2009
Li 2011^44^	1.75 (1.16 to 2.63)	0.007	N/A	European, Asian	1001	1889	N/A	Walsh 2007, Terry 2008, Ashton 2009, Nunobiki 2009, Ueda 2009
Knappskog 2012^40^	1.22 (1.03 to 1.44)	N/A	0.36	European	392	956	N/A	Hospital based
Zajac 2012^27^	1.33 (1.12 to 1.58)	0.001	N/A	European	152	100	N/A	Hospital based
Yoneda 2013^52^	1.64 (0.81 to 3.28)	0.450	0.45	Asian	125	200	All	Population based
Peng 2013^41^	1.6 (1.21 to 2.13)	0.001	N/A	European, Asian	2069	4546	N/A	Walsh 2007, Terry 2008, Ashton 2009, Nunobiki 2009, Knappskog 2012, Yoneda 2013
	1.87 (1.29 to 2.73)	0.010	N/A	European	1842	4251	N/A	
Zhao 2014^53^	1.41 (1.04 to 1.92)	0.030	N/A	European, Asian	1278	2189	N/A	Walsh 2007, Terry 2008, Ashton 2009, Ueda 2009, Zajac 2012, Yoneda 2013
	1.34 (1.07 to 1.69)	N/A	N/A	European	859	1707	N/A	
Wang 2014^38^	1.32 (1.06 to 1.64)	0.010	N/A	European, Asian	1967	4460	N/A	Walsh 2007, Terry 2008, Ashton 2009, Nunobiki 2009, Ueda 2009, Zajac 2012, Knappskog 2012, Yoneda 2013
	1.14 (0.79 to 1.65)	0.490	N/A	European	1769	4172	N/A	
Xue 2016^42^	1.46 (1.25 to 1.72)	N/A	N/A	European	1690	4151	N/A	Walsh 2007, Terry 2008, Ashton 2009, Nunobiki 2009, Ueda 2009, Zajac 2012, Knappskog 2012, Yoneda 2013
Zhang 2018^54^	1.91 (1.5 to 3.47)	0.035	N/A	European, Asian	762	1041	N/A	Walsh 2007, Terry 2008, Ashton 2009, Nunobiki 2009, Ueda 2009, Zajac 2012
Zou 2018^55^	1.23 (1.06 to 1.41)	0.005	N/A	European, Asian, mixed	3535	6476	All	Walsh 2007, Terry 2008, Ashton 2009, Ueda 2009, Knappskog 2012, Zajac 2012, Yoneda 2013, Okamoto 2015, Gansmo 2017^37^

*Walsh *et al* 2007 and Gansmo *et al* 2017 did not meet eligibility criteria for us to include in our evaluation.

EAF, effect allele frequency; EC, endometrial cancer; SNP, single nucleotide polymorphism.

In comparison, a GWAS including nearly 13 000 cases found no evidence of an association with OR and corresponding 95% CI of 1.00 (0.97 to 1.03) and a p value of 0.93 (personal communication).^21^ Nevertheless, we cannot completely rule out a role for *MDM2* variants in endometrial cancer predisposition as the candidate-gene studies reported larger effects in Asians, whereas the GWAS primarily contained participants of European ancestry. There is also some suggestion that the SNP309 variant is in linkage disequilibrium with another variant, SNP285, which confers an opposite effect.

It is worth noting that the SNP285C/SNP309G haplotype frequency was observed in up to 8% of Europeans, thus requiring correction for the confounding effect of SNP285C in European studies.^39^ However, aside from one study conducted by Knappskog *et al*, no other study including the meta-analyses corrected for the confounding effect of SNP285.^40^ Among the studies presented in table 3, Knappskog *et al* (2012) reported that after correcting for SNP285, the OR for association of this haplotype with endometrial cancer was much lower, though still significant. Unfortunately, the meta-analyses which synthesised Knappskog *et al* (2012), as part of their analysis, did not correct for SNP285C in the European-based studies they included.^38 41 42^ It is also concerning that two meta-analyses using the same primary articles failed to report the same result, in two instances.^38 42–44^

## Discussion

This article represents the most comprehensive systematic review to date, regarding critical appraisal of the available evidence of common low-penetrance variants implicated in predisposition to endometrial cancer. We have identified the most robust SNPs in the context of endometrial cancer risk. Of those, only 19 were significant at genome-wide level and a further five were considered marginally significant. The largest GWAS conducted in this field was the discovery- and meta-GWAS by O’Mara *et al*, which utilised 12 096 cases and 108 979 controls.^21^ Despite the inclusion of all published GWAS and around 5000 newly genotyped cases, the total number did not reach anywhere near what is currently available for other common cancers such as breast cancer. For instance, BCAC (Breast Cancer Association Consortium) stands at well over 200 000 individuals with more than half being cases, and resulted in identification of ~170 SNPs in relation to breast cancer.^19 45^ A total of 313 SNPs including imputations were then used to derive a PRS for breast cancer.^19^ Therefore, further efforts should be directed to recruit more patients, with deep phenotypic clinical data to allow for relevant adjustments and subgroup analyses to be conducted for better precision.

A recent pre-print study by Zhang and colleagues examined the polygenicity and potential for SNP-based risk prediction for 14 common cancers, including endometrial cancer, using available summary-level data from European-ancestry datasets.^46^ They estimated that there are just over 1000 independent endometrial cancer susceptibility SNPs, and that a PRS comprising all such SNPs would have an area under the receiver-operator curve of 0.64, similar to that predicted for ovarian cancer, but lower than that for the other cancers in the study. The modelling in the paper suggests that an endometrial cancer GWAS double the size of the current largest study would be able to identify susceptibility SNPs together explaining 40% of the genetic variance, but that in order to explain 75% of the genetic variance it would be necessary to have a GWAS comprising close to 150 000 cases and controls, far in excess of what is currently feasible.

We found that the literature consists mainly of candidate-gene studies with small sample sizes, meta-analyses reporting conflicting results despite using the same set of primary articles, and multiple reports of significant SNPs that have not been validated by any larger GWAS. The candidate-gene studies were indeed the most useful and cheaper technique available until the mid to late 2000s. However, a lack of reproducibility (particularly due to population stratification and reporting bias), uncertainty of reported associations, and considerably high false discovery rates make these studies much less appropriate in the post-GWAS era. Unlike the candidate-gene approach, GWAS do not require prior knowledge, selection of genes or SNPs, and provide vast amounts of data. Furthermore, both the genotyping process and data analysis phases have become cheaper, the latter particularly due to faster and open-access pre-phasing and imputation tools being made available.

It is clear from table 1 that some SNPs were reported with wide 95% CI, which can be directly attributed to small sample sizes particularly when restricting the cases to non-endometrioid histology only, low EAF or poor imputation quality. Thus, these should be interpreted with caution. Additionally, most of the SNPs reported by candidate-gene studies were not detected by the largest GWAS to date conducted by O’Mara *et al*.^21^ However, this does not necessarily mean that the possibility of those SNPs being relevant should be completely dismissed. Moreover, meta-analyses were attempted for other variants; however, these showed no statistically significant association and many presented with high heterogeneity between the respective studies (data not shown). Furthermore, as many studies utilised the same set of cases and/or controls, conducting a meta-analysis was not possible for a good number of SNPs. It is therefore unequivocal that the literature is crowded with numerous small candidate-gene studies and conflicting data. This makes it particularly hard to detect novel SNPs and conduct meaningful meta-analyses.

We found convincing evidence for 19 variants that indicated the strongest association with endometrial cancer, as shown in table 1. The associations between endometrial cancer and variants in or around *HNF1B*, *CYP19A1*, *SOX4*, *MYC*, *KLF* and *EIF2AK* found in earlier GWAS were then replicated in the latest and largest GWAS. These SNPs showed promising potential in a theoretical PRS we devised based on published data. Using all 24 or genome-wide significant SNPs only, women with a PRS in the top 1% of the distribution would be predicted to have a risk of endometrial cancer 3.16 and 2.09 times higher than the mean risk, respectively.

However, the importance of these variants and relevance of the proximate genes in a functional or biological context is challenging to evaluate. Long distance promoter regulation by enhancers may disguise the genuine target gene. In addition, enhancers often do not loop to the nearest gene, further complicating the relevance of nearby gene(s) to a GWAS hit. In order to elucidate biologically relevant candidate target genes in endometrial cancer, O’Mara *et al* looked into promoter-associated chromatin looping using a modern HiChIP approach.^47^ The authors utilised normal and tumoural endometrial cell lines for this analysis which showed significant enrichment for endometrial cancer heritability, with 103 candidate target genes identified across the 13 risk loci identified by the largest ECAC GWAS. Notable genes identified here were *CDKN2A* and *WT1*, and their antisense counterparts. The former was reported to be nearby of rs1679014 and the latter of rs10835920, as shown in table 1. Moreover, of the 36 candidate target genes, 17 were found to be downregulated while 19 were upregulated in endometrial tumours.

The authors also investigated overlap between the 13 endometrial cancer risk loci and top eQTL variants for each target gene.^47^ In whole blood, of the two particular lead SNPs, rs8822380 at 17q21.32 was a top eQTL for *SNX11* and *HOXB2*, whereas rs937213 at 15q15.1 was a top eQTL for *SRP14*. In endometrial tumour, rs7579014 at 2p16.1 was found to be a top eQTL for *BCL11A*. This is particularly interesting because *BCL11A* was the only nearby/candidate gene that had a GWAS association reported in both endometrioid and non-endometrioid subtypes. The study looked at protein–protein interactions between endometrial cancer drivers and candidate target gene products. Significant interactions were observed with TP53 (most significant), AKT, PTEN, ESR1 and KRAS, among others. Finally, when 103 target candidate genes and 387 proteins were combined together, 462 pathways were found to be significantly enriched. Many of these are related to gene regulation, cancer, obesity, insulinaemia and oestrogen exposure. This study clearly showed a potential biological relevance for some of the SNPs reported by ECAC GWAS in 2018.

Most of the larger included studies used cohorts primarily composed of women of broad European descent. Hence, there are negligible data available for other ethnicities, particularly African women. This is compounded by the lack of reference genotype data available for comparative analysis, making it harder for research to be conducted in ethnicities other than Europeans. This poses a problem for developing risk prediction models that are equally valuable and predictive across populations. Thus, our results also are of limited applicability to non-European populations.

Furthermore, considering that non-endometrioid cases comprise a small proportion (~20%) of all endometrial cancer cases, much larger cohort sizes are needed to detect any genuine signals for non-endometrioid tumours. Most of the evaluated studies looked at either overall/mixed endometrial cancer subtypes or endometrioid histology, and those that looked at variant associations with non-endometrioid histology were unlikely to have enough power to detect any signal with statistical significance. This is particularly concerning because non-endometrioid subtypes are biologically aggressive tumours with a much poorer prognosis that contribute disproportionately to mortality from endometrial cancer. It is particularly important that attempts to improve early detection and prevention of endometrial cancer focus primarily on improving outcomes from these subtypes. It is also worth noting that, despite the current shift towards a molecular classification of endometrial cancer, most studies used the overarching classical Bokhman’s classification system, type I versus type II, or no histological classification system at all. Therefore, it is important to create and follow a standardised and comprehensive classification system for reporting tumour subtypes for future studies.

This study compiled and presented available information for an extensively studied, yet unproven in large datasets, SNP309 variant in *MDM2*. Currently, there is no convincing evidence for an association between this variant and endometrial cancer risk. Additionally, of all the studies, only one accounted for the opposing effect of a nearby variant SNP285 in their analyses. Thus, we conclude that until confirmed by a sufficiently large GWAS, this variant should not be considered significant in influencing the risk of endometrial cancer and therefore not included in a PRS. This is also true for the majority of the SNPs reported in candidate-gene studies, as the numbers fall far short of being able to detect genuine signals.

This systematic review presents the most up-to-date evidence for endometrial cancer susceptibility variants, emphasising the need for further large-scale studies to identify more variants of importance, and validation of these associations. Until data from larger and more diverse cohorts are available, the top 24 SNPs presented here are the most robust common genetic variants that affect endometrial cancer risk. The multiplicative effects of these SNPs could be used in a PRS to allow personalised risk prediction models to be developed for targeted screening and prevention interventions for women at greatest risk of endometrial cancer.

## References

[1] Sundar S, Balega J, Crosbie E, Drake A, Edmondson R, Fotopoulou C, Gallos I, Ganesan R, Gupta J, Johnson N, Kitson S, Mackintosh M, Martin-Hirsch P, Miles T, Rafii S, Reed N, Rolland P, Singh K, Sivalingam V, Walther A BGCS uterine cancer guidelines: recommendations for practice. Eur J Obstet Gynecol Reprod Biol 2017;213:71–97. 10.1016/j.ejogrb.2017.04.015 28437632

[2] Siegel RL, Miller KD, Jemal A Cancer statistics, 2019. CA Cancer J Clin 2019;69:7–34. 10.3322/caac.21551 30620402

[3] Morice P, Leary A, Creutzberg C, Abu-Rustum N, Darai E Endometrial cancer. Lancet 2016;387:1094–108. 10.1016/S0140-6736(15)00130-0 26354523

[4] Tzur T, Kessous R, Weintraub AY Current strategies in the diagnosis of endometrial cancer. Arch Gynecol Obstet 2017;296:5–14. 10.1007/s00404-017-4391-z 28508342

[5] Clarke MA, Devesa SS, Harvey SV, Wentzensen N Hysterectomy-corrected uterine corpus cancer incidence trends and differences in relative survival reveal racial disparities and rising rates of nonendometrioid cancers. J Clin Oncol 2019;37:1895–908. 10.1200/JCO.19.00151 31116674 PMC6675596

[6] Kandoth C, Schultz N, Cherniack AD, Akbani R, Liu Y, Shen H, Robertson AG, Pashtan I, Shen R, Benz CC, Yau C, Laird PW, Ding L, Zhang W, Mills GB, Kucherlapati R, Mardis ER, Levine DA Getz G, Gabriel SB, Cibulskis K, Lander E, Sivachenko A, Sougnez C, Lawrence M, Dooling D, Fulton R, Fulton L, Kalicki-Veizer J, McLellan MD, O'Laughlin M, Schmidt H, Wilson RK, Ye K, Ally A, Balasundaram M, Birol I, Butterfield YSN, Carlsen R, Carter C, Chu A, Chuah E, Chun HJE, Dhalla N, Guin R, Hirst C, Holt RA, Jones SJM, Lee D, HI L, Marra MA, Mayo M, Moore RA, Mungall AJ, Plettner P, Schein JE, Sipahimalani P, Tam A, Varhol RJ, Saksena G, Onofrio RC, Schumacher SE, Tabak B, Carter SL, Hernandez B, Gentry J, Salvesen HB, Ardlie K, Winckler W, Beroukhim R, Meyerson M, Hadjipanayis A, Lee S, Mahadeshwar HS, Park P, Protopopov A, Ren XJ, Seth S, Song XZ, Tang JB, RB X, Yang LX, Zeng D, Chin L, Zhang JH, Auman JT, Balu S, Bodenheimer T, Buda E, Hayes DN, Hoyle AP, Jefferys SR, Jones CD, Meng SW, Mieczkowski PA, Mose LE, Parker JS, Perou CM, Roach J, Shi Y, Simons JV, Soloway MG, Tan DH, Topal MD, Waring S, JY W, Hoadley KA, Baylin SB, Bootwalla MS, Lai PH, Triche TJ, Van Den Berg D, Weisenberger DJ, Cho J, DiCara D, Frazer S, Heiman D, Jing R, Lin P, Mallard W, Stojanov P, Voet D, Zhang HL, Zou LH, Noble M, Reynolds SM, Shmulevich I, Aksoy BA, Antipin Y, Ciriello G, Dresdner G, Gao JJ, Gross B, Jacobsen A, Ladanyi M, Reva B, Sander C, Sinha R, Sumer SO, Taylor BS, Cerami E, Weinhold N, Benz S, Goldstein T, Haussler D, Ng S, Szeto C, Stuart J, Annala M, Broom BM, Casasent TD, ZL J, Liang H, YL L, Unruh AK, Wakefield C, Weinstein JN, Zhang NX, Liu YX, Broaddus R, Adams C, Barr T, Black AD, Bowen J, Deardurff J, Frick J, Gastier-Foster JM, Grossman T, Harper HA, Hart-Kothari M, Helsel C, Hobensack A, Kuck H, Kneile K, Leraas K, Lichtenberg TM, McAllister C, Pyatt RE, Ramirez NC, Tabler TR, Vanhoose N, White P, Wise L, Zmuda E, Barnabas N, Berry-Green C, Blanc V, Boice L, Button M, Farkas A, Green A, MacKenzie J, Nicholson D, Kalloger SE, Gilks CB, Karlan BY, Lester J, Orsulic S, Borowsky M, Cadungog M, Czerwinski C, Huelsenbeck-Dill L, Iacocca M, Petrelli N, Witkin G, Nemirovich-Danchenko E, Potapova O, Rotin D, Berchuck A, Birrer M, DiSaia P, Monovich L, Curley E, Gardner J, Mallery D, Penny R, Dowdy SC, Winterhoff B, Dao L, Gostout B, Meuter A, Teoman A, Dao F, Olvera N, Bogomolniy F, Garg K, Soslow RA, Abramov M, Bartlett JMS, Kodeeswaran S, Parfitt J, Moiseenko F, Clarke BA, Goodman MT, Carney ME, Matsuno RK, Fisher J, Huang M, Rathmell WK, Thorne L, Van Le L DR, Edwards R, Elishaev E, Zorn K, Goodfellow PJ, Mutch D, Kahn AB, Bell DW, Pollock PM, Wang C, Wheeler DA, Shinbrot E, Ayala B, Chu AL, Jensen MA, Kothiyal P, Pihl TD, Pontius J, Pot DA, Snyder EE, Srinivasan D, Shaw KRM, Sheth M, Davidsen T, Eley G, Ferguson ML, Demchok JA, Yang LM, Guyer MS, Ozenberger BA, Sofia HJ, Shen R, Cancer Genome Atlas Research Network Integrated genomic characterization of endometrial carcinoma. Nature 2013;497:67–73. 10.1038/nature12113 23636398 PMC3704730

[7] Stelloo E, Bosse T, Nout RA, MacKay HJ, Church DN, Nijman HW, Leary A, Edmondson RJ, Powell ME, Crosbie EJ, Kitchener HC, Mileshkin L, Pollock PM, Smit VT, Creutzberg CL Refining prognosis and identifying targetable pathways for high-risk endometrial cancer; a TransPORTEC initiative. Mod Pathol 2015;28:836–44. 10.1038/modpathol.2015.43 25720322

[8] Win AK, Reece JC, Ryan S Family history and risk of endometrial cancer: a systematic review and meta-analysis. Obstet Gynecol 2015;125:89–98. 10.1097/AOG.0000000000000563 25560109

[9] Constantinou P, Tischkowitz M Genetics of gynaecological cancers. Best Pract Res Clin Obstet Gynaecol 2017;42:114–24. 10.1016/j.bpobgyn.2017.01.004 28202331

[10] Ryan NAJ, Glaire MA, Blake D, Cabrera-Dandy M, Evans DG, Crosbie EJ The proportion of endometrial cancers associated with Lynch syndrome: a systematic review of the literature and meta-analysis. Genet Med 2019. 10.1038/s41436-019-0536-8 PMC8076013 31086306

[11] Lichtenstein P, Holm NV, Verkasalo PK, Iliadou A, Kaprio J, Koskenvuo M, Pukkala E, Skytthe A, Hemminki K Environmental and heritable factors in the causation of cancer — analyses of cohorts of twins from Sweden, Denmark, and Finland. N Engl J Med 2000;343:78–85. 10.1056/NEJM200007133430201 10891514

[12] Lu Y, Ek WE, Whiteman D, Vaughan TL, Spurdle AB, Easton DF, Pharoah PD, Thompson DJ, Dunning AM, Hayward NK, Chenevix-Trench G, Macgregor S, Q-MEGA and AMFS Investigators, ANECS-SEARCH, UKOPS-SEARCH, BEACON Consortium Most common 'sporadic' cancers have a significant germline genetic component. Hum Mol Genet 2014;23:6112–8. 10.1093/hmg/ddu312 24943595 PMC4271103

[13] Kitson SJ, Evans DG, Crosbie EJ Identifying high-risk women for endometrial cancer prevention strategies: proposal of an endometrial cancer risk prediction model. Cancer Prev Res 2017;10:1–13. 10.1158/1940-6207.CAPR-16-0224 27965288

[14] Wan YL, Beverley-Stevenson R, Carlisle D, Clarke S, Edmondson RJ, Glover S, Holland J, Hughes C, Kitchener HC, Kitson S, Miles T, Morley R, Morrison J, Nelson L, Powell M, Sadler L, Tomlinson A, Tylko-Hill K, Whitcombe J, Crosbie EJ Working together to shape the endometrial cancer research agenda: the top ten unanswered research questions. Gynecol Oncol 2016;143:287–93. 10.1016/j.ygyno.2016.08.333 27593736

[15] Raglan O, Kalliala I, Markozannes G, Cividini S, Gunter MJ, Nautiyal J, Gabra H, Paraskevaidis E, Martin-Hirsch P, Tsilidis KK, Kyrgiou M Risk factors for endometrial cancer: an umbrella review of the literature. Int J Cancer 2019;145:1719–30. 10.1002/ijc.31961 30387875

[16] PROSPERO Low-penetrance common predisposition variants in endometrial cancer risk: a systematic review, 2018.

[17] VonVille H Excel workbooks for systematic reviews, 2015 Available: https://showcase.dropbox.com/s/Excel-Workbooks-for-Systematic-Reviews-User-Guides-Kf4pYVTvFqSJdZR2Spzlu [Accessed 17 Oct 2017].

[18] Spencer CCA, Su Z, Donnelly P, Marchini J Designing genome-wide association studies: sample size, power, imputation, and the choice of genotyping CHIP. PLoS Genet 2009;5:13 10.1371/journal.pgen.1000477 PMC2688469 19492015

[19] Mavaddat N, Michailidou K, Dennis J, Lush M, Fachal L, Lee A, Tyrer JP, Chen T-H, Wang Q, Bolla MK, Yang X, Adank MA, Ahearn T, Aittomäki K, Allen J, Andrulis IL, Anton-Culver H, Antonenkova NN, Arndt V, Aronson KJ, Auer PL, Auvinen P, Barrdahl M, Beane Freeman LE, Beckmann MW, Behrens S, Benitez J, Bermisheva M, Bernstein L, Blomqvist C, Bogdanova NV, Bojesen SE, Bonanni B, Børresen-Dale A-L, Brauch H, Bremer M, Brenner H, Brentnall A, Brock IW, Brooks-Wilson A, Brucker SY, Brüning T, Burwinkel B, Campa D, Carter BD, Castelao JE, Chanock SJ, Chlebowski R, Christiansen H, Clarke CL, Collée JM, Cordina-Duverger E, Cornelissen S, Couch FJ, Cox A, Cross SS, Czene K, Daly MB, Devilee P, Dörk T, Dos-Santos-Silva I, Dumont M, Durcan L, Dwek M, Eccles DM, Ekici AB, Eliassen AH, Ellberg C, Engel C, Eriksson M, Evans DG, Fasching PA, Figueroa J, Fletcher O, Flyger H, Försti A, Fritschi L, Gabrielson M, Gago-Dominguez M, Gapstur SM, García-Sáenz JA, Gaudet MM, Georgoulias V, Giles GG, Gilyazova IR, Glendon G, Goldberg MS, Goldgar DE, González-Neira A, Grenaker Alnæs GI, Grip M, Gronwald J, Grundy A, Guénel P, Haeberle L, Hahnen E, Haiman CA, Håkansson N, Hamann U, Hankinson SE, Harkness EF, Hart SN, He W, Hein A, Heyworth J, Hillemanns P, Hollestelle A, Hooning MJ, Hoover RN, Hopper JL, Howell A, Huang G, Humphreys K, Hunter DJ, Jakimovska M, Jakubowska A, Janni W, John EM, Johnson N, Jones ME, Jukkola-Vuorinen A, Jung A, Kaaks R, Kaczmarek K, Kataja V, Keeman R, Kerin MJ, Khusnutdinova E, Kiiski JI, Knight JA, Ko Y-D, Kosma V-M, Koutros S, Kristensen VN, Krüger U, Kühl T, Lambrechts D, Le Marchand L, Lee E, Lejbkowicz F, Lilyquist J, Lindblom A, Lindström S, Lissowska J, Lo W-Y, Loibl S, Long J, Lubiński J, Lux MP, MacInnis RJ, Maishman T, Makalic E, Maleva Kostovska I, Mannermaa A, Manoukian S, Margolin S, Martens JWM, Martinez ME, Mavroudis D, McLean C, Meindl A, Menon U, Middha P, Miller N, Moreno F, Mulligan AM, Mulot C, Muñoz-Garzon VM, Neuhausen SL, Nevanlinna H, Neven P, Newman WG, Nielsen SF, Nordestgaard BG, Norman A, Offit K, Olson JE, Olsson H, Orr N, Pankratz VS, Park-Simon T-W, Perez JIA, Pérez-Barrios C, Peterlongo P, Peto J, Pinchev M, Plaseska-Karanfilska D, Polley EC, Prentice R, Presneau N, Prokofyeva D, Purrington K, Pylkäs K, Rack B, Radice P, Rau-Murthy R, Rennert G, Rennert HS, Rhenius V, Robson M, Romero A, Ruddy KJ, Ruebner M, Saloustros E, Sandler DP, Sawyer EJ, Schmidt DF, Schmutzler RK, Schneeweiss A, Schoemaker MJ, Schumacher F, Schürmann P, Schwentner L, Scott C, Scott RJ, Seynaeve C, Shah M, Sherman ME, Shrubsole MJ, Shu X-O, Slager S, Smeets A, Sohn C, Soucy P, Southey MC, Spinelli JJ, Stegmaier C, Stone J, Swerdlow AJ, Tamimi RM, Tapper WJ, Taylor JA, Terry MB, Thöne K, Tollenaar RAEM, Tomlinson I, Truong T, Tzardi M, Ulmer H-U, Untch M, Vachon CM, van Veen EM, Vijai J, Weinberg CR, Wendt C, Whittemore AS, Wildiers H, Willett W, Winqvist R, Wolk A, Yang XR, Yannoukakos D, Zhang Y, Zheng W, Ziogas A, Dunning AM, Thompson DJ, Chenevix-Trench G, Chang-Claude J, Schmidt MK, Hall P, Milne RL, Pharoah PDP, Antoniou AC, Chatterjee N, Kraft P, García-Closas M, Simard J, Easton DF, Freeman LEB, Alnaes GIG, YD K, WY L, Kostovska IM, Rennert HS, Clarke C, Balleine R, Baxter R, Braye S, Carpenter J, Dahlstrom J, Forbes J, Lee CS, Marsh D, Morey A, Pathmanathan N, Scott R, Simpson P, Spigelman A, Wilcken N, Yip D, Zeps N, Sexton A, Dobrovic A, Christian A, Trainer A, Fellows A, Shelling A, De Fazio A, Blackburn A, Crook A, Meiser B, Patterson B, Saunders C, Hunt C, Amor D, Ortega DG, Edkins E, Salisbury E, Haan E, Macrea F, Farshid G, Lindeman G, Trench G, Mann G, Giles G, Gill G, Thorne H, Campbell I, Hickie I, Caldon L, Winship I, Cui J, Flanagan J, Kollias J, Visvader J, Taylor J, Burke J, Saunus J, Hopper J, Beesley J, Kirk J, French J, Tucker K, Wu K, Phillips K, Forrest L, Lipton L, Andrews L, Lobb L, Walker L, Kentwell M, Spurdle M, Cummings M, Gleeson M, Harris M, Jenkins M, Young MA, Delatycki M, Wallis M, Burgess M, Brown M, Southey M, Bogwitz M, Field M, Friedlander M, Gattas M, Saleh M, Aghmesheh M, Hayward N, Pachter N, Cohen P, Duijf P, James P, Fong P, Butow P, Williams R, Kefford R, Dawson SJ, Lok S, O'Connell S, Greening S, Nightingale S, Edwards S, Fox S, McLachlan SA, Lakhani S, Dudding T, Antill Y, Sahlberg KK, Ottestad L, Karesen R, Schlichting E, Holmen MM, Sauer T, Haakensen V, Engebraten O, Naume B, Fossa A, Kiserud CE, Reinertsen KV, Helland A, Riis M, Geisler J, ABCTB Investigators, kConFab/AOCS Investigators, NBCS Collaborators Polygenic risk scores for prediction of breast cancer and breast cancer subtypes. Am J Hum Genet 2019;104:21–34. 10.1016/j.ajhg.2018.11.002 30554720 PMC6323553

[20] Geng YH, Wang ZF, Jia YM, Zheng LY, Chen L, Liu DG, XH L, Tian XX, Fang WG Genetic polymorphisms in CDH1 are associated with endometrial carcinoma susceptibility among Chinese Han women. Oncol Lett 2018;16:6868–78. 30405831 10.3892/ol.2018.9469 PMC6202459

[21] O’Mara TA, Glubb DM, Amant F, Annibali D, Ashton K, Attia J, Auer PL, Beckmann MW, Black A, Bolla MK, Brauch H, Brenner H, Brinton L, Buchanan DD, Burwinkel B, Chang-Claude J, Chanock SJ, Chen C, Chen MM, Cheng THT, Clarke CL, Clendenning M, Cook LS, Couch FJ, Cox A, Crous-Bous M, Czene K, Day F, Dennis J, Depreeuw J, Doherty JA, Dörk T, Dowdy SC, Dürst M, Ekici AB, Fasching PA, Fridley BL, Friedenreich CM, Fritschi L, Fung J, García-Closas M, Gaudet MM, Giles GG, Goode EL, Gorman M, Haiman CA, Hall P, Hankison SE, Healey CS, Hein A, Hillemanns P, Hodgson S, Hoivik EA, Holliday EG, Hopper JL, Hunter DJ, Jones A, Krakstad C, Kristensen VN, Lambrechts D, Marchand LL, Liang X, Lindblom A, Lissowska J, Long J, Lu L, Magliocco AM, Martin L, McEvoy M, Meindl A, Michailidou K, Milne RL, Mints M, Montgomery GW, Nassir R, Olsson H, Orlow I, Otton G, Palles C, Perry JRB, Peto J, Pooler L, Prescott J, Proietto T, Rebbeck TR, Risch HA, Rogers PAW, Rübner M, Runnebaum I, Sacerdote C, Sarto GE, Schumacher F, Scott RJ, Setiawan VW, Shah M, Sheng X, Shu X-O, Southey MC, Swerdlow AJ, Tham E, Trovik J, Turman C, Tyrer JP, Vachon C, VanDen Berg D, Vanderstichele A, Wang Z, Webb PM, Wentzensen N, Werner HMJ, Winham SJ, Wolk A, Xia L, Xiang Y-B, Yang HP, Yu H, Zheng W, Pharoah PDP, Dunning AM, Kraft P, De Vivo I, Tomlinson I, Easton DF, Spurdle AB, Thompson DJ Identification of nine new susceptibility loci for endometrial cancer. Nat Commun 2018;9 10.1038/s41467-018-05427-7 PMC6085317 30093612

[22] Painter JN, Kaufmann S, O'Mara TA, Hillman KM, Sivakumaran H, Darabi H, Cheng THT, Pearson J, Kazakoff S, Waddell N, Hoivik EA, Goode EL, Scott RJ, Tomlinson I, Dunning AM, Easton DF, French JD, Salvesen HB, Pollock PM, Thompson DJ, Spurdle AB, Edwards SL A common variant at the 14q32 endometrial cancer risk locus activates Akt1 through YY1 binding. Am J Hum Genet 2016;98:1159–69. 10.1016/j.ajhg.2016.04.012 27259051 PMC4908177

[23] Spurdle AB, Thompson DJ, Ahmed S, Ferguson K, Healey CS, O'Mara T, Walker LC, Montgomery SB, Dermitzakis ET, Fahey P, Montgomery GW, Webb PM, Fasching PA, Beckmann MW, Ekici AB, Hein A, Lambrechts D, Coenegrachts L, Vergote I, Amant F, Salvesen HB, Trovik J, Njolstad TS, Helland H, Scott RJ, Ashton K, Proietto T, Otton G, Tomlinson I, Gorman M, Howarth K, Hodgson S, Garcia-Closas M, Wentzensen N, Yang H, Chanock S, Hall P, Czene K, Liu J, Li J, Shu X-O, Zheng W, Long J, Xiang Y-B, Shah M, Morrison J, Michailidou K, Pharoah PD, Dunning AM, Easton DF, Australian National Endometrial Cancer Study Group, National Study of Endometrial Cancer Genetics Group Genome-wide association study identifies a common variant associated with risk of endometrial cancer. Nat Genet 2011;43:451–4. 10.1038/ng.812 21499250 PMC3770523

[24] O'Mara TA, Glubb DM, Painter JN, Cheng T, Dennis J, Attia J, Holliday EG, McEvoy M, Scott RJ, Ashton K, Proietto T, Otton G, Shah M, Ahmed S, Healey CS, Gorman M, Martin L, Hodgson S, Fasching PA, Hein A, Beckmann MW, Ekici AB, Hall P, Czene K, Darabi H, Li J, Dürst M, Runnebaum I, Hillemanns P, Dörk T, Lambrechts D, Depreeuw J, Annibali D, Amant F, Zhao H, Goode EL, Dowdy SC, Fridley BL, Winham SJ, Salvesen HB, Njølstad TS, Trovik J, Werner HMJ, Tham E, Liu T, Mints M, Bolla MK, Michailidou K, Tyrer JP, Wang Q, Hopper JL, Peto J, Swerdlow AJ, Burwinkel B, Brenner H, Meindl A, Brauch H, Lindblom A, Chang-Claude J, Couch FJ, Giles GG, Kristensen VN, Cox A, Pharoah PDP, Dunning AM, Tomlinson I, Easton DF, Thompson DJ, Spurdle AB, Australian National Endometrial Cancer Study Group (ANECS), National Study of Endometrial Cancer Genetics Group (NSECG), RENDOCAS, AOCS Group Comprehensive genetic assessment of the ESR1 locus identifies a risk region for endometrial cancer. Endocr Relat Cancer 2015;22:851–61. 10.1530/ERC-15-0319 26330482 PMC4559752

[25] Chen MM, Crous-Bou M, Setiawan VW, Prescott J, Olson SH, Wentzensen N, Black A, Brinton L, Chen C, Chen C, Cook LS, Doherty J, Friedenreich CM, Hankinson SE, Hartge P, Henderson BE, Hunter DJ, Le Marchand L, Liang X, Lissowska J, Lu L, Orlow I, Petruzella S, Polidoro S, Pooler L, Rebbeck TR, Risch H, Sacerdote C, Schumacher F, Sheng X, Shu X-ou, Weiss NS, Xia L, Van Den Berg D, Yang HP, Yu H, Chanock S, Haiman C, Kraft P, De Vivo I, Vivo D Exome-wide association study of endometrial cancer in a multiethnic population. PLoS One 2014;9:9 10.1371/journal.pone.0097045 PMC4014590 24810602

[26] Cheng THT, Thompson D, Painter J, O’Mara T, Gorman M, Martin L, Palles C, Jones A, Buchanan DD, Win AK, Hopper J, Jenkins M, Lindor NM, Newcomb PA, Gallinger S, Conti D, Schumacher F, Casey G, Giles GG, Pharoah P, Peto J, Cox A, Swerdlow A, Couch F, Cunningham JM, Goode EL, Winham SJ, Lambrechts D, Fasching P, Burwinkel B, Brenner H, Brauch H, Chang-Claude J, Salvesen HB, Kristensen V, Darabi H, Li J, Liu T, Lindblom A, Hall P, de Polanco ME, Sans M, Carracedo A, Castellvi-Bel S, Rojas-Martinez A, Aguiar Jnr S, Teixeira MR, Dunning AM, Dennis J, Otton G, Proietto T, Holliday E, Attia J, Ashton K, Scott RJ, McEvoy M, Dowdy SC, Fridley BL, Werner HMJ, Trovik J, Njolstad TS, Tham E, Mints M, Runnebaum I, Hillemanns P, Dörk T, Amant F, Schrauwen S, Hein A, Beckmann MW, Ekici A, Czene K, Meindl A, Bolla MK, Michailidou K, Tyrer JP, Wang Q, Ahmed S, Healey CS, Shah M, Annibali D, Depreeuw J, Al-Tassan NA, Harris R, Meyer BF, Whiffin N, Hosking FJ, Kinnersley B, Farrington SM, Timofeeva M, Tenesa A, Campbell H, Haile RW, Hodgson S, Carvajal-Carmona L, Cheadle JP, Easton D, Dunlop M, Houlston R, Spurdle A, Tomlinson I Meta-analysis of genome-wide association studies identifies common susceptibility polymorphisms for colorectal and endometrial cancer near SH2B3 and TSHZ1. Sci Rep 2015;5:12 10.1038/srep17369 PMC4664893 26621817

[27] Zając A, Stachowiak G, Pertyński T, Romanowicz H, Wilczyński J, Smolarz B Association between MDM2 SNP309 polymorphism and endometrial cancer risk in Polish women. Pjp 2012;4:278–83. 10.5114/pjp.2012.32776 23359199

[28] Lundin E, Wirgin I, Lukanova A, Afanasyeva Y, Krogh V, Axelsson T, Hemminki K, Clendenen TV, Arslan AA, Ohlson N, Sieri S, Roy N, Koenig KL, Idahl A, Berrino F, Toniolo P, Hallmans G, Försti A, Muti P, Lenner P, Shore RE, Zeleniuch-Jacquotte A Selected polymorphisms in sex hormone-related genes, circulating sex hormones and risk of endometrial cancer. Cancer Epidemiol 2012;36:445–52. 10.1016/j.canep.2012.04.006 22633539 PMC3663487

[29] Tao MH, Cai Q, Zhang Z-F, Xu W-H, Kataoka N, Wen W, Xiang Y-B, Zheng W, Shu XO Polymorphisms in the CYP19A1 (aromatase) gene and endometrial cancer risk in Chinese women. Cancer Epidemiol Biomarkers Prev 2007;16:943–9. 10.1158/1055-9965.EPI-06-1012 17507620

[30] Cheng TH, Thompson DJ, O'Mara TA, Painter JN, Glubb DM, Flach S, Lewis A, French JD, Freeman-Mills L, Church D, Gorman M, Martin L, Hodgson S, Webb PM, Attia J, Holliday EG, McEvoy M, Scott RJ, Henders AK, Martin NG, Montgomery GW, Nyholt DR, Ahmed S, Healey CS, Shah M, Dennis J, Fasching PA, Beckmann MW, Hein A, Ekici AB, Hall P, Czene K, Darabi H, Li J, Dörk T, Dürst M, Hillemanns P, Runnebaum I, Amant F, Schrauwen S, Zhao H, Lambrechts D, Depreeuw J, Dowdy SC, Goode EL, Fridley BL, Winham SJ, Njølstad TS, Salvesen HB, Trovik J, Werner HM, Ashton K, Otton G, Proietto T, Liu T, Mints M, Tham E, Consortium C, Jun Li M, Yip SH, Wang J, Bolla MK, Michailidou K, Wang Q, Tyrer JP, Dunlop M, Houlston R, Palles C, Hopper JL, Peto J, Swerdlow AJ, Burwinkel B, Brenner H, Meindl A, Brauch H, Lindblom A, Chang-Claude J, Couch FJ, Giles GG, Kristensen VN, Cox A, Cunningham JM, Pharoah PDP, Dunning AM, Edwards SL, Easton DF, Tomlinson I, Spurdle AB, National Study of Endometrial Cancer Genetics Group (NSECG), Australian National Endometrial Cancer Study Group (ANECS), RENDOCAS, AOCS Group Five endometrial cancer risk loci identified through genome-wide association analysis. Nat Genet 2016;48:667–74. 10.1038/ng.3562 27135401 PMC4907351

[31] O'Mara TA, Glubb DM, Kho PF, Thompson DJ, Spurdle AB Genome-wide association studies of endometrial cancer: latest developments and future directions. Cancer Epidemiol Biomarkers Prev 2019;28:1095–102. 10.1158/1055-9965.EPI-18-1031 31040137

[32] Banno K, Yanokura M, Iida M, Masuda K, Aoki D Carcinogenic mechanisms of endometrial cancer: involvement of genetics and epigenetics. J Obstet Gynaecol Res 2014;40:1957–67. 10.1111/jog.12442 25131761

[33] Lacey JV, Yang H, Gaudet MM, Dunning A, Lissowska J, Sherman ME, Peplonska B, Brinton LA, Healey CS, Ahmed S, Pharoah P, Easton D, Chanock S, Garcia-Closas M Endometrial cancer and genetic variation in PTEN, PIK3CA, Akt1, MLH1, and MSH2 within a population-based case-control study. Gynecol Oncol 2011;120:167–73. 10.1016/j.ygyno.2010.10.016 21093899 PMC3073848

[34] Chen MM, O'Mara TA, Thompson DJ, Painter JN, Attia J, Black A, Brinton L, Chanock S, Chen C, Cheng TH, Cook LS, Crous-Bou M, Doherty J, Friedenreich CM, Garcia-Closas M, Gaudet MM, Gorman M, Haiman C, Hankinson SE, Hartge P, Henderson BE, Hodgson S, Holliday EG, Horn-Ross PL, Hunter DJ, Le Marchand L, Liang X, Lissowska J, Long J, Lu L, Magliocco AM, Martin L, McEvoy M, Olson SH, Orlow I, Pooler L, Prescott J, Rastogi R, Rebbeck TR, Risch H, Sacerdote C, Schumacher F, Wendy Setiawan V, Scott RJ, Sheng X, Shu X-O, Turman C, Van Den Berg D, Wang Z, Weiss NS, Wentzensen N, Xia L, Xiang Y-B, Yang HP, Yu H, Zheng W, Pharoah PDP, Dunning AM, Tomlinson I, Easton DF, Kraft P, Spurdle AB, De Vivo I, LG L, Setiawan VW, Vivo D, Australian National Endometrial Cancer Study Group (ANECS), National Study Of Endometrial Cancer Genetics Group (NSECG) GWAS meta-analysis of 16 852 women identifies new susceptibility locus for endometrial cancer. Hum Mol Genet 2016;25:ddw092–20. 10.1093/hmg/ddw092 PMC5868213 27008869

[35] Setiawan VW, Haessler J, Schumacher F, Cote ML, Deelman E, Fesinmeyer MD, Henderson BE, Jackson RD, Vöckler J-S, Wilkens LR, Yasmeen S, Haiman CA, Peters U, Le Marchand L, Kooperberg C Hnf1B and endometrial cancer risk: results from the page study. PLoS One 2012;7:6 10.1371/journal.pone.0030390 PMC3267708 22299039

[36] Walsh CS, Miller CW, Karlan BY, Koeffler HP Association between a functional single nucleotide polymorphism in the MDM2 gene and sporadic endometrial cancer risk. Gynecol Oncol 2007;104:660–4. 10.1016/j.ygyno.2006.10.008 17123590

[37] Gansmo LB, Bjørnslett M, Halle MK, Salvesen HB, Romundstad P, Hveem K, Vatten L, Dørum A, Lønning PE, Knappskog S Mdm2 promoter polymorphism del1518 (rs3730485) and its impact on endometrial and ovarian cancer risk. BMC Cancer 2017;17:6 10.1186/s12885-017-3094-y 28158999 PMC5291962

[38] Wang L-H, Wang X, Xu W-T, Hu Y-L Mdm2 rs2279744 polymorphism and endometrial cancer: a meta-analysis. Tumor Biol 2014;35:3167–70. 10.1007/s13277-013-1413-8 24293392

[39] Knappskog S, Gansmo LB, Dibirova K, Metspalu A, Cybulski C, Peterlongo P, Aaltonen L, Vatten L, Romundstad P, Hveem K, Devilee P, Evans GD, Lin D, Van Camp G, Manolopoulos VG, Osorio A, Milani L, Ozcelik T, Zalloua P, Mouzaya F, Bliznetz E, Balanovska E, Pocheshkova E, Kučinskas V, Atramentova L, Nymadawa P, Titov K, Lavryashina M, Yusupov Y, Bogdanova N, Koshel S, Zamora J, Wedge DC, Charlesworth D, Dörk T, Balanovsky O, Lønning PE Population distribution and ancestry of the cancer protective MDM2 SNP285 (rs117039649). Oncotarget 2014;5:8223–34. 10.18632/oncotarget.1910 25327560 PMC4226679

[40] Knappskog S, Trovik J, Marcickiewicz J, Tingulstad S, Staff AC, Romundstad P, Hveem K, Vatten L, Salvesen HB, Lønning PE SNP285C modulates oestrogen receptor/Sp1 binding to the MDM2 promoter and reduces the risk of endometrial but not prostatic cancer. Eur J Cancer 2012;48:1988–96. 10.1016/j.ejca.2011.10.024 22119201

[41] Peng Q, Mo C, Qin A, Lao X, Chen Z, Sui J, Wu J, Zhai L, Yang S, Qin X, Li S Mdm2 SNP309 polymorphism contributes to endometrial cancer susceptibility: evidence from a meta-analysis. J Exp Clin Cancer Res 2013;32 10.1186/1756-9966-32-85 PMC4029393 24423195

[42] Xue Z, Zhu X, Teng Y Relationship between murine double minute 2 (MDM2) T309G polymorphism and endometrial cancer risk: a meta-analysis. Med Sci Monit 2016;22:3186–90. 10.12659/MSM.896973 27604213 PMC5026055

[43] Wan Y, Wu W, Yin Z, Guan P, Zhou B Mdm2 SNP309, gene-gene interaction, and tumor susceptibility: an updated meta-analysis. BMC Cancer 2011;11:9 10.1186/1471-2407-11-208 21619694 PMC3115916

[44] Li Y, Zhao H, Sun L, Huang L, Yang Q, Kong B Mdm2 SNP309 is associated with endometrial cancer susceptibility: a meta-analysis. Hum Cell 2011;24:57–64. 10.1007/s13577-011-0013-4 21547352

[45] Hamdi Y, Soucy P, Adoue V, Michailidou K, Canisius S, Lemaçon A, Droit A, Andrulis IL, Anton-Culver H, Arndt V, Baynes C, Blomqvist C, Bogdanova NV, Bojesen SE, Bolla MK, Bonanni B, Borresen-Dale A-L, Brand JS, Brauch H, Brenner H, Broeks A, Burwinkel B, Chang-Claude J, Couch FJ, Cox A, Cross SS, Czene K, Darabi H, Dennis J, Devilee P, Dörk T, Dos-Santos-Silva I, Eriksson M, Fasching PA, Figueroa J, Flyger H, García-Closas M, Giles GG, Goldberg MS, González-Neira A, Grenaker-Alnæs G, Guénel P, Haeberle L, Haiman CA, Hamann U, Hallberg E, Hooning MJ, Hopper JL, Jakubowska A, Jones M, Kabisch M, Kataja V, Lambrechts D, Le Marchand L, Lindblom A, Lubinski J, Mannermaa A, Maranian M, Margolin S, Marme F, Milne RL, Neuhausen SL, Nevanlinna H, Neven P, Olswold C, Peto J, Plaseska-Karanfilska D, Pylkäs K, Radice P, Rudolph A, Sawyer EJ, Schmidt MK, Shu X-O, Southey MC, Swerdlow A, Tollenaar RAEM, Tomlinson I, Torres D, Truong T, Vachon C, Van Den Ouweland AMW, Wang Q, Winqvist R, Zheng W, Benitez J, Chenevix-Trench G, Dunning AM, Pharoah PDP, Kristensen V, Hall P, Easton DF, Pastinen T, Nord S, Simard J, Collaborators N, Truong Th, Ouweland AMWVD, Easton A, Pastinen T, Nord S, Simard J, Couch FJ, NBCS Collaborators, kConFab/AOCS Investigators Association of breast cancer risk with genetic variants showing differential allelic expression: identification of a novel breast cancer susceptibility locus at 4q21. Oncotarget 2016;7:80140–63. 10.18632/oncotarget.12818 27792995 PMC5340257

[46] Zhang Y, Wilcox A, Zhang H, Choudhury PP, Easton D, Milne R, Simard J, Hall P, Michailidou K, Dennis J, Schmidt M, Chang-Claude J, Gharahkhani P, Whiteman D, Campbell P, Hoffmeister M, Jenkins M, Peters U, Hsu L, Gruber S, Casey G, Schmit S, O’Mara T, Spurdle A, Thompson D, Tomlinson I, Vivo D, Landi MT, Law M, Iles M, Demenais F, Kumar R, MacGregor S, Bishop T, Ward S, Bondy M, Houlston R, Wiencke J, Melin B, Barnholtz-Sloan J, Kinnersley B, Wrensch M, Amos C, Hung R, Brennan P, McKay J, Caporaso N, Berndt S, Birmann B, Camp N, Kraft P, Rothman N, Slager S, Berchuck A, Pharoah PDP, Sellers T, Gayther S, Pearce C, Goode E, Schildkraut J, Moysich K, Amundadottir L, Jacobs E, Klein A, Petersen G, Risch H, Stolzenberg-Solomon R, Wolpin B, Li D, Eeles R, Haiman C, Kote-Jarai Z, Schumacher F, Olama AAA, Purdue M, Scelo G, Dalgaard M, Greene M, Grotmol T, Kanetsky P, McGlynn K, Nathanson K, Turnbull C, Wiklund F, Chanock S, Chatterjee N, Garcia-Closas M, Bcac B, Ccfr C, Ecac G, GenoMel G, Ilcco I, InterLymph O, Oral Cancer G, Panc P Assessment of polygenic architecture and risk prediction based on common variants across fourteen cancers. bioRxiv 2019. 10.1038/s41467-020-16483-3 PMC7335068 32620889

[47] O’Mara TA, Spurdle AB, Glubb DM, Endometrial Cancer Association Consortium Endometrial Cancer Association Consortium Analysis of promoter-associated chromatin interactions reveals biologically relevant candidate target genes at endometrial cancer risk loci. Cancers 2019;11:1440 10.3390/cancers11101440 PMC6826789 31561579

[48] Terry K, McGrath M, Lee I-M, Buring J, De Vivo I, Vivo D Mdm2 SNP309 is associated with endometrial cancer risk. Cancer Epidemiol Biomarkers Prev 2008;17:983–6. 10.1158/1055-9965.EPI-07-2872 18398041 PMC2728574

[49] Ashton KA, Proietto A, Otton G, Symonds I, McEvoy M, Attia J, Gilbert M, Hamann U, Scott RJ Polymorphisms in TP53 and MDM2 combined are associated with high grade endometrial cancer. Gynecol Oncol 2009;113:109–14. 10.1016/j.ygyno.2008.12.036 19193430

[50] Nunobiki O, Ueda M, Yamamoto M, Toji E, Sato N, Izuma S, Okamoto Y, Torii K, Noda S Polymorphisms of p53 codon 72 and MDM2 promoter 309 and the risk of endometrial cancer. Hum Cell 2009;22:101–6. 10.1111/j.1749-0774.2009.00075.x 19874399

[51] Ueda M, Yamamoto M, Nunobiki O, Toji E, Sato N, Izuma S, Okamoto Y, Torii K, Noda S Murine double-minute 2 homolog single nucleotide polymorphism 309 and the risk of gynecologic cancer. Hum Cell 2009;22:49–54. 10.1111/j.1749-0774.2009.00068.x 19379464

[52] Yoneda T, Kuboyama A, Kato K, Ohgami T, Okamoto K, Saito T, Wake N Association of MDM2 SNP309 and TP53 Arg72Pro polymorphisms with risk of endometrial cancer. Oncol Rep 2013;30:25–34. 10.3892/or.2013.2433 23624782 PMC3729233

[53] Zhao Y, Yang X, Hao X, Pan X, Zhao B, Ma J, Fang J, Zhao M Common variant on MDM2 contributes to endometrial cancer susceptibility: evidence based on 7 studies. Tumor Biol 2014;35:7555–60. 10.1007/s13277-014-1886-0 24792886

[54] Zhang JX, Zhang Y, Zhang ZY Association of rs2279744 and rs117039649 promoter polymorphism with the risk of gynecological cancer a meta-analysis of case-control studies. Medicine 2018;97:9. 10.1097/MD.0000000000009554 PMC5943879 29480845

[55] Zou XW, Zhang Y, Zhang L, JX L, Zhu CJ, Cheng QH, Zhou JH, Chen YG Association between MDM2 SNP309 and endometrial cancer risk a PRISMA-compliant meta-analysis. Medicine 2018;97:11. 10.1097/MD.0000000000013273 PMC6310604 30544386

